# Patient with recurrence dermatofibrosarcoma protuberans: A case report

**DOI:** 10.1002/ccr3.2868

**Published:** 2020-04-16

**Authors:** Mohammadreza Amjadzadeh, Parvin Mousavi Ghanavati

**Affiliations:** ^1^ Student Research Committee Ahvaz Jundishapur University of Medical Sciences Ahvaz Iran

**Keywords:** dermatofibrosarcoma, recurrence, surgery, tumor

## Abstract

We present a typical case of dermatofibrosarcoma protuberans(DFSP) with a complaint of a painful and various recurrence, make a review of the most important aspects. This is a rare tumor and we call special attention to the fact that its recurrence is extremely frequent, so there is absolute need to observe these patients periodically after surgery.

## Graphical abstract

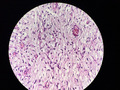

## 1 INTRODUCTION

Dermatofibrosarcoma is a malignant skin tumor that accounts for <0.1% of all malignant neoplasms and 1% of all soft tissue sarcomas. It is a relatively uncommon soft tissue neoplasm of fibroblast origin. Slow growth and low aggressiveness are its characteristics. Its incidence is 0.8‐4.1 per million people annually.^1^, ^2^ The prevalence of the disease is higher in women than in men (4.4% vs 4.2% per million people per year). The highest prevalence of dermatofibrosarcoma protuberans(DFSP) is in the age group of 20‐50. The tumor has a local invasion, and a high recurrence rate (10%‐60%), that is due to the highly irregular shape and finger protrusions of the tumor, but rarely causes the regional or distant metastases.^3^, ^4^ The trunk (42%‐72%) and extremities (20%‐30%) and head and neck (10%‐16%) areas are the most common DFSP sites. These tumors occur mostly in areas such as surgical scar sites, chronic burns, traumatic sites, common vascular sites, vaccine injection sites, insect bites, and radiation‐induced dermatitis.^5^, ^6^, ^7^, ^8^

## 2 CASE PRESENTATION

We present the case of a 22‐year‐old Caucasian patient who had referred to an outpatient clinic with a complaint of a painful and recurrent mass in the right axillary region. First, the patient reported an increasing size of the tumor during the preceding 6 months and denied any recent weight loss, fever, night sweats, or chills. There was neither personal nor familial history of malignancy but he had a lot of times of recurrency in the first refer. The patient underwent mass resection, but he does not have any pathologic report. The first recurrency was in 2013, the patient presented with a recurrence of the tumor in the previous area. The mass has a cystic heterogeneous of 29.20.30 mm and a volume of 9.5 cm^3^. The patient underwent mass resection. The pathologic diagnosis of the extracted specimen was nodular fasciitis. The second recurrence was in 2016, the patient returned with a complaint of a painful mass in the right axillary region and underwent surgery—the pathologic result of the resected sample diagnosed as benign soft tissue tumor most compatible with myoxid fibroma. The following year, the patient presented with a similar painful mass in the same area where the ultrasound of the right axillary region had shown an isoechoic mass of 41.39 mm in the subcutaneous area. The patient underwent surgery again, and the removed mass sent for pathologic examination. The report shows myxoid fibroma recurrence.

**Figure 1:**
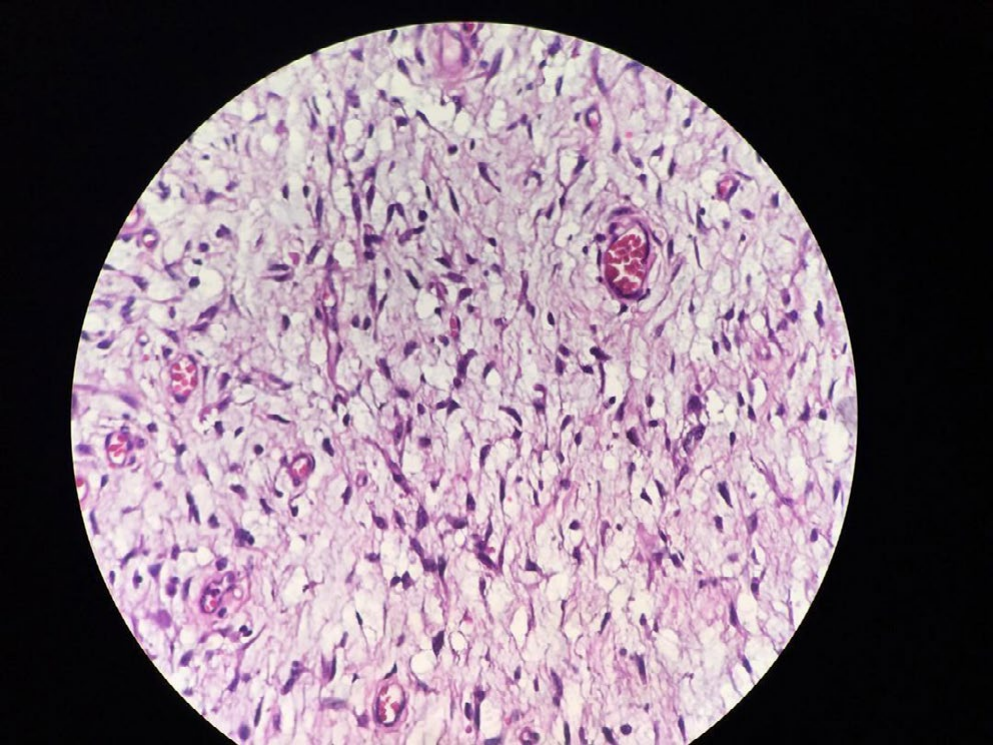
Characteristic storiform pattern seen in patient with DFSP

Eventually,

In 2019, patients presenting with painful recurrence has the same complaint and also in the same area as the previous ultrasound, which displays a hypoechoic area of approximately 9.6.8 mm with a depth of 8 mm. Also, a lymph node of hypoechoic density (4.5 × 8 mm) observed adjacent to the lesion. Highly suspicious two hypoechoic nodules of 17 × 8 and 6 × 24 mm were seen in the right axillary region, suggesting a recurrent tumor invasion to the adjacent nodal lymph. Axillary tumor of the patient underwent FNA. The extracted specimens examined for pathology and IHC and according to pathologic findings and IHC, DFSP diagnosis made for the patient. The patient underwent mass resection for the last time, and the resected mass sent for pathological examination. The diagnosis results in myxoid form of DFSP. Also, the extracted specimen re‐examined by IHC where the tumor cells tested positive for SMA and B‐carenin, a part of tumor is positive for CD34 negative for S100, thereby setting the diagnosis of DFSP tumor markers were confirmed again and finally, the diagnosis approved as fibrosarcoma originating from DFSP (Figure 1).

## 3 DISCUSSION

DFSP is a rare and locally aggressive dermal mesenchymal neoplasm. Frierson and Cooper first reported the rare DFSP myxoid variants in 1983.^7^This type of neoplasm is also referred to as the cutaneous origin. It is responsible for <0.1% of all malignancies and approximately 1% of all soft tissue sarcomas. An annual incidence rate of DFSP is 0.8‐4.5 cases per million individuals.^1^

DFSP usually develops as purple, pink, and reddish‐brown plaques on the surface of the skin and gradually becomes multiple "protuberant" nodules. This lesion is highly predisposed to recurrence due to its high capacity for infiltration into the underlying tissues, including the subcutaneous and facial tissues, and the muscles and bones.^2^, ^3^, ^4^, ^5^, ^6^, ^7^, ^8^

In our case, the lesion was restricted to the skin and fascia and did not invade the bones and muscles.

According to Martin et al, half of the patients with DFSP initially present with a "nonprotuberant" lesion, and on average after 7.6‐9.3 years, these lesions become protuberant DFSP. In the early stages, we consider differential diagnoses, including lipomas, epidermal cysts, keloids, dermatofibroma, and nodular fasciitis. If the patient presents in the late stages, the lesion may be mistaken for pyogenic granuloma, Kaposi sarcoma, and other soft tissue sarcoma.^1^

Recent studies of DFSP in the head and neck regions are much more invasive than the trunk. There have also been reports of metastasis to the lung, which has been extremely rare. The mass in our study was restricted only to the right axilla and the surrounding lymph nodes. We found no evidence of metastasis.

Studies have also found the utility of imaging modalities such as magnetic resonance imaging (MRI), computed tomography (CT), and ultrasound to be useful in preoperative evaluations.^3^

Typical findings in ultrasound studies show hypoechoic or mixed hyperechoic, with mostly well‐defined margins or irregularities, but our ultrasound findings initially show an isoechoic mass with defined margins and subsequent recurrence of the lesions presented with hypoechoic defined margins mass.

The gold standard to diagnose this type of neoplasm is a deep subcutaneous punch biopsy or incisional biopsy because the surface biopsy of the lesion cannot differentiate sufficiently from benign skin lesions. Re‐biopsy is recommended if our clinical suspicion was strong and unequivocal primary biopsy was performed although this is associated with a risk of tumor seeding.

Histopathological studies usually show relatively uniform densely grouped fusiform cells, with elongated nuclei and without significant cytologic atypia or pleomorphism in characteristic storiform arrangement.

Immunohistochemically, tumor cells stain for vimentin, CD34, apolipoprotein D, nestin, and maybe for EMA. Desmin, S100 protein, FXIIIa, stromelysin III, HMGA1 & 2, tenascin, D2‐40, CD163, and keratins are negative. In myoid nodules, tumor cells stain for SMA. Fibro sarcomatous DFSP may exhibit a loss of CD34 and enhancing expression of TP53^9,20,21^. In our case, tumor cells were positive for SMA and B‐catenin and part of tumor was positive for CD34 and negative for S100. As a result, the findings strongly favor DFSP.

The American Joint Committee did not publish a TNM staging system since we used a system presented by Ugurel et al to evaluate the situation includes stage‐I—localized disease, stage‐II—lymph node metastasis, and stage‐III—distal metastasis.

Our patient had a stage II disease because of lymph node metastasis and no distant metastasis.

The standard treatment of localized masses is extensive local surgical resection with 2‐3 cm surgical margins and three‐dimensional resection of skin, subcutaneous tissue, and underlying fascia.

The local recurrence rate tends to decrease with the increase in surgical margins. An alternative to extensive surgical resection is Mohs micrographic surgery which has a therapeutic success rate of 93%‐100%.^3^

Chemotherapy is not the standard treatment for this disease, but recent studies with Imatinib mesylate as Neoadjuvant have recommended in advanced and recurrent DFSP localized tumors. The recommended dose is 800 mg orally, which has also been approved by the FDA.^4^

After surgery, we recommended the patient to chemotherapy because of the recurrence and large volume of the lining mass. Recent chemotherapy is considered to be a wise choice based on patient concerns about recurrence, cosmetic problems, dysfunction, and even metastasis. In one study, the rate of cure in these patients following postoperative chemotherapy was 85%. However, recurrences usually occur in the first 3 years, half of which usually occur 1 year after surgery, which illustrates the importance of regular check‐ups.

The NCCN guidelines recommend ongoing follow‐up with a focus on the lesion site every 6‐12 months, with a re‐biopsy of any suspicious symptoms or signs.

## 4 CONCLUSIONS

This is a rare tumor and we call special attention to the fact that its recurrence is extremely frequent, so there is absolute need to observe these patients periodically after surgery. This case motivated us to review the most important aspects of this rare tumor, besides presenting an interesting and typical case of DFSP with recurrence. All dermatologists and surgeons must know about the frequent recurrence of this tumor, sometimes even when excised with wide margins. For this reason, these patients must be observed periodically after surgery for a long time.

## CONFLICT OF INTEREST

Authors report no conflict of interest.

## AUTHOR CONTRIBUTIONS

All authors wrote and edited the manuscript equally.
